# Investigation of Spatial Coupling Coordination Development: Identifying Land System States from the Adaptation–Conflict Perspective

**DOI:** 10.3390/ijerph20010373

**Published:** 2022-12-26

**Authors:** Xingjia Wang, Dongyan Wang, Wanying Gao, Jiaxi Lu, Xiaotong Jin

**Affiliations:** College of Earth Sciences, Jilin University, Changchun 130061, China

**Keywords:** human–environment system, ecosystem service balance, land use conflict, coupling coordination degree, spatio-temporal differences

## Abstract

With the advancement of global urbanization, ecosystem conservation and sustainable land development have become major issues. In this context, the uncoordinated and imbalanced development of the land-centered human–environment system requires urgent attention, especially in rust belt cities that pose critical challenges to regional land system sustainability. Therefore, taking Changchun City from 1990 to 2020 as an example, we identified and evaluated the ecosystem service (ES) balance and land use conflict from the perspectives of internal support and external development pressure. Based on the land system adaptation and conflict results, a coupling coordination degree model (CCDM) was constructed to investigate the spatio-temporal characteristics of land system development. The results indicated that there was an obvious downward trend in the regional ES balance, while areas with higher ES balance were mainly distributed in the eastern ecologically sound areas, and southern built-up areas presented deficient ES balance levels (i.e., demand exceeding supply), with a significant expansion trend from 1990 to 2020. Land use conflict was especially prominent in areas experiencing rapid rural–urban transformation, and the hot spots expanded noticeably. The spatio-temporal differences in the coupling coordination degree of ES balance and land use conflict were significant, whereas the land system of the study area has always been dominated by a balanced development pattern throughout the research period, except for the urban center, which tended to be in a stage of uncoordinated development, with the ES balance blocked. These findings suggest that it is necessary to coordinate urban and adjacent regions through regionally integrated efforts to alleviate the ES imbalance. This research can provide a scientific reference for analyzing regional land system states, coordinating the sustainable spatial development of ecosystems, and implementing revitalization strategies to achieve win-win land system goals.

## 1. Introduction

The United Nations’ 2030 Agenda for Sustainable Development presents ambitious targets for global sustainability, aiming to advance the coupled and coordinated development of multiple factors of human society and the geographical environment. This is particularly the case in the context of Goal 15, which emphasizes the targets of “protect, restore, and promote sustainable use of terrestrial ecosystems” and “stop and reverse the land degradation phenomenon” [1]. Numerous countries and regions have witnessed an unavoidable aggravation of inharmonious and unbalanced man–land relationships resulting from rapid urbanization, industrialization processes, and their linkages of natural resource systems and environmental bases [2,3,4]. These phenomena, mainly in terms of high population growth and rampant land expansion, have had implications for the rising demand for compatible spaces and ecosystem services (ESs), which, in turn, results in prominent social problems, resource depletion, land use conflicts, and ecosystem deterioration and will inevitably continue to pose substantial threats to the sustainability of the human–environment system [5]. It is generally believed that ES studies can provide reliable theoretical support for ecological and environmental management, with regional ES balance being perceived as the comprehensive embodiment of ecosystem health, which essentially reveals the spatial adaptation of the environment conditions and utilities that ecosystems form and that maintain the survival and development of human beings [6,7]. A sound supply–demand pattern of the regional ecosystem guarantees the orderly operation of social activities and maximizes human welfare. Therefore, understanding the spatio-temporal evolutions of ES balance is the necessary prerequisite for revealing the differences between the supply and demand of ESs and is critical in identifying ecological problems, which is essential for accessing resource benefits and drafting sustainable management plans [5,8,9,10]. In recent decades, research related to ES balance has made great progress. Due to the differences in the ES capacity of different land use types, the one-to-one correspondence matrix between land use/land cover (LULC) and ES balance has been proven to be an effective method for quantifying regional ES balance [8,11,12].

As a prominent manifestation of the land system development process, the unsustainability of the natural environment under the pressure of anthropogenic processes makes the land use structure appear to be an uncoordinated development [13]. The existence of spatial heterogeneity and temporal lags between land development and socio-economics advancement forms pressure differences between internal and external land systems, driving the land system development in the direction of hyper-social adaptation [14], and thus comes the growing interest in land use conflicts. In particular, identifying regional land use conflicts, analyzing their spatio-temporal patterns, and formulating reconciliation schemes are topics receiving continuous attention in land use studies [15,16]. There are numerous ways to conceptualize and evaluate land use conflict spatially, such as the multi-criteria evaluation method, value compatibility analysis, pressure–state–response models, and landscape ecological risk assessment, among others [17,18]. Some scholars argue that land use conflict occurs when land use resources are in a disharmonious state because of the multiple negative externalities between the land use space and human activities [19,20,21]. Since land use conflict is a microcosm of socio-economic contexts, it has been accepted not only as a sensitive indicator of human–environment interactions but also as a major barrier to human–environment sustainability [22,23]. Thus, it is crucial to select appropriate methods to map the spatial characteristics of regional land use conflict under different land development processes in order to scientifically regulate and guide land system conflicts.

At present, sustainable land management is considered a key issue of regional development and is on many agendas. The acceleration of development demands has resulted in the constraining role of land resources and the natural environment in human production. To the best of our knowledge, nearly two-thirds of global ecosystems are being subjected to degradation, whereas land use/cover change (LUCC) is regarded as one of the most significant elements influencing the direction of present and future changes in ESs [24]. Due to continuously increasing land use pressure, the ecosystem must improve its impact resistance, adaptive capacity, and resilience, and promote the positive migration of ES balance, to enable the resolution and absorption of land use conflict disturbance [25,26]. China is now facing a new situation of the unified management of natural resources and the integration of territorial spatial planning. It is imperative, therefore, to give more attention to land system management and to promote the overall planning of multiple factors so as to guide decision making regarding natural resource management and planning. The land system is recognized as a complex adaptive system embedded in the “socio-ecological” system. Whether the human–environment relationships or the socio-ecological systems are integrated subjects in balancing the complicated relationships between adaptation and conflict, the key point that is frequently emphasized is what is the internal logical underlying the land system, ES balance, and land use conflict? Existing studies have shown that land system changes impact ES balance and land use conflict by transforming the land use structure and functions [27]. However, these previous studies have mainly focused on land system change as a basic driving force for ES conversion and man–land spatial contradictions. As mentioned previously, it is clear that both ES balance and land use conflict essentially reflect the sustainable effect between economic development, social stability, and environmental health, which explicitly manifests as spatial competition for land-centered resources and adaptation processes among various entities and stakeholders. So why not integrate ES balance and land use conflict as the internal support and external development pressure of a land system to reflect its development status?

Additionally, in the context of global development transformation and territorial spatial planning, how to attribute the problem to the spatial linkage between sustainable land use development and the flow of ES supply and demand, how to investigate the spatio-temporal development characteristics of a land system, and how to provide operational guidance based on the spatial planning system for regions where the ES balance is poor and has a lagging transformation level are still issues of great significance [28,29]. Scholars and policymakers who are focusing on the sustainable development of land systems have begun to find a harmonious state of development, in which the coupling coordination degree model (CCDM) is deemed to be an adequate method to investigate the complicated eco-environment and land use issues [30]. Since the future land system is bound to change constantly, exploring its coupling and coordination from the “adaptation (ES balance)–conflict (land use conflict)” perspective will contribute to improving the connotation of land use sustainability.

In this study, Changchun City was chosen as the study case, which has undergone fast growth and recessions, has lost abundant natural habitats, and is haunted by significant regional imbalance [31]. The internal support and external development pressure of the land system in this region have evolved in response to the changing relationship between land resources and human development needs. Therefore, evaluating the state of the land system from the “adaptation–conflict” perspective is an effective means to grasp the spatial arrangement and coordination development patterns of this region. The aim of this study is to deal with the new trade-offs between ecological sustainability and land use development to provide sufficient space for long-term objectives by focusing on the revitalization of rust belt cities in the postindustrial era of China and to try to address the following targets: (a) to effectively assess the spatial patterns and changes in ES balance from 1990 to 2020, (b) to identify and analyze the spatio-temporal evolution and regional differentiation of land use conflict between 1990 and 2020, and (c) to quantitatively examine the coupling coordination relationships of ES balance and land use conflict and to track land system states from 1990 to 2020. This research will provide a theoretical basis for promoting the coordinated development of land systems and thereby render reliable information to guide land management, help decision makers manage and reconcile the conflicts over land use structure and ecosystem conservation, and contribute to the task of sustainable development.

## 2. Materials and Methods

### 2.1. Research Area

**Figure 1:**
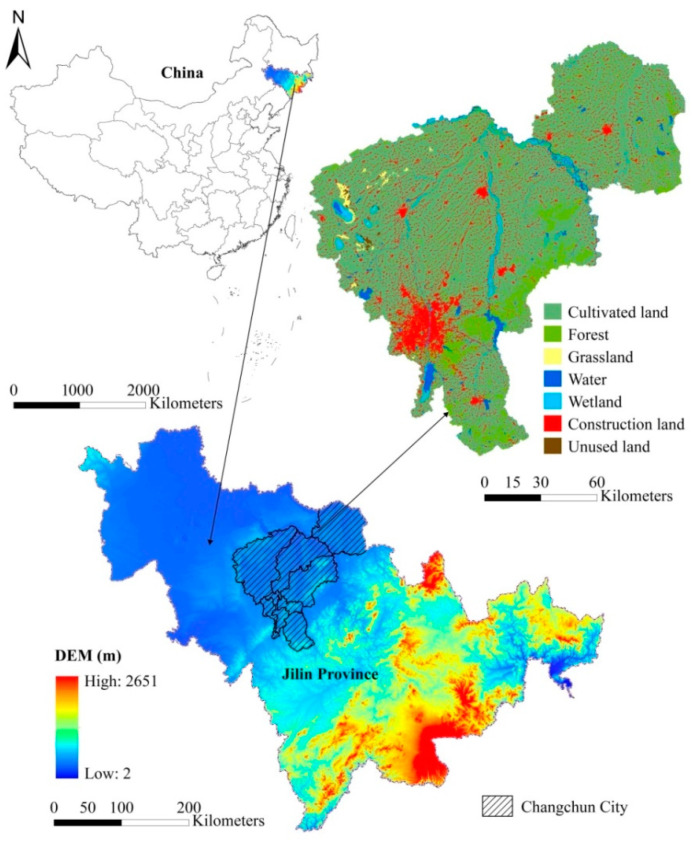
Geographic location of the study area and spatial pattern of land use in 2020.

Changchun City, which is situated in the middle of the Northeast China Plain, is the central city of the Northeast Asian Economic Circle and the capital of Jilin Province, China (Figure 1). It comprises 10 counties (county-level cities) and districts with diverse land use types, covering a total area of 20,594.86 km^2^. As an important industrial base and national comprehensive transportation hub, it has witnessed a noteworthy expansion in its urban area and rapid industrialization in recent years, making Changchun a city with a profound modern heritage. Consequently, various land use conflicts have arisen in Changchun City, especially those involving the quantity and spatial structures of land use, resource endowments, and landscape patterns. Benefiting from abundant natural resources and fertile soil conditions, Changchun City is also a natural eco-environmental system and a crucial base for grain production that contributes enormously to national life. Land use transformation and policy-led land use patterns potentially affect the demands and supplies of ESs. Like most rust belt cities, Changchun City is grappling with the perennial contradiction between the man–land relationship and the environmental basis of the land system. Therefore, the prominent uncertainty of the coordinated development between urban economic development and ESs in this region has made Changchun City an ideal area for measuring the dynamics of ES balance under the complex land use conflicts that arise during the process of urban renewal and green development.

### 2.2. Data Sources and Processing

**Figure 2:**
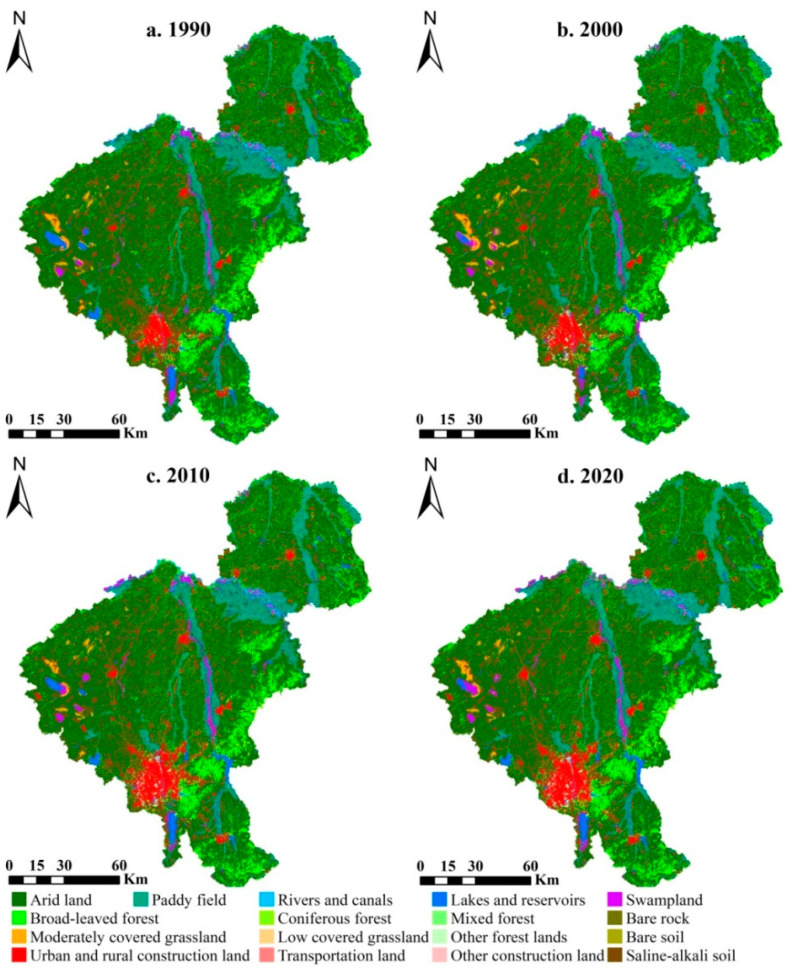
Map of the LULC patterns in Changchun City in 1990 (**a**), 2000 (**b**), 2010 (**c**), and 2020 (**d**), respectively.

The LULC data used in this study were derived from Landsat TM/ETM+/OLI remote sensing images of 1990, 2000, 2010, and 2020, including 7 first-level land use categories (i.e., cultivated land, forest, grassland, water, wetland, construction land, and unused land) and 17 second-level types (Figure 2), with a spatial resolution of 30 m. Various data sources, including field surveys, historical records, and Google Earth maps, were used to verify, correct, and settle the multi-temporal LULC data. The overall accuracy of artificial visual interpretation results with the assistance of machine learning was no less than 90%, and the kappa coefficients in 1990, 2000, 2010, and 2020 were 0.83, 0.86, 0.94, and 0.87, respectively. Considering the scale of the study area, spatial LULC accuracy, calculation workload, and visualization, the 3 km fishnet was selected as the basic spatial analysis unit through comparative analysis at multiple grid scales, which fully conformed with the usage requirements for small-scale assessments of land use conflict and ES balance.

### 2.3. Assessment Methods

#### 2.3.1. Quantitative Assessments of ES Supply, Demand, and Balance

**Figure 3:**
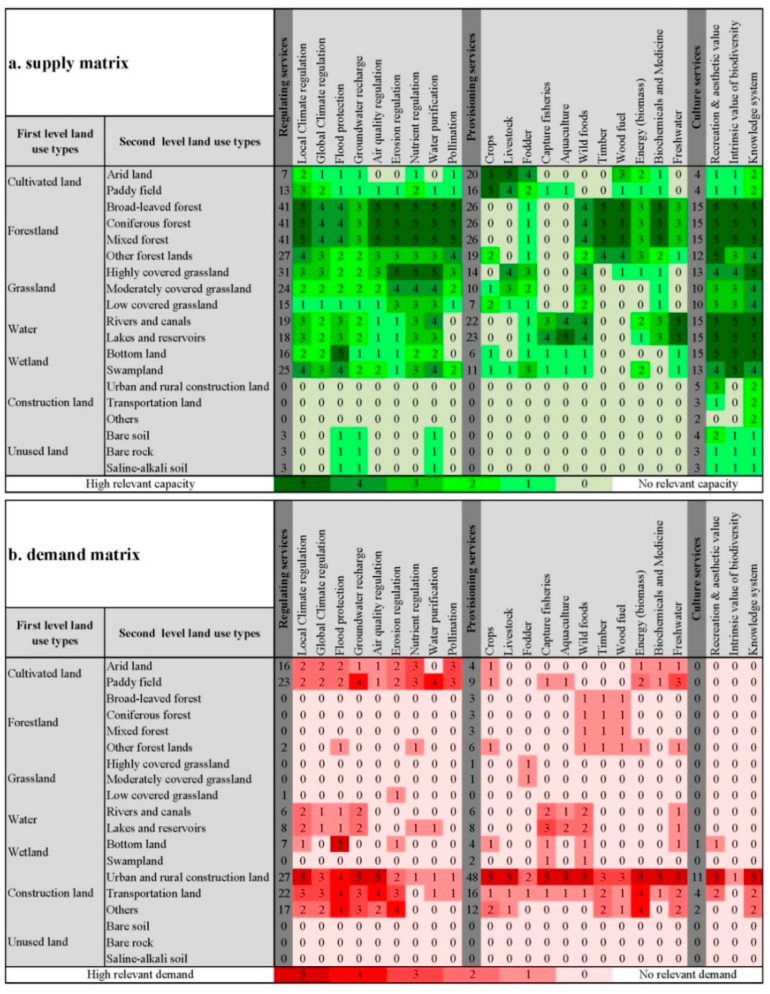
Assessment matrices illustrating the supply capacity (**a**) and demand (**b**) for ecosystem services of different land use types in Changchun City, China. Due to the differences between the land cover classification systems, we integrated the LULC matrices of previous relevant studies [5,33], with experts assessing the construction of the supply–demand matrices suitable for this study. The values indicate the following capacities/demands: 0 = no relevant capacity/demand; 1 = low relevant capacity/demand; 2 = relevant capacity/demand; 3 = medium capacity/demand; 4 = high relevant capacity/demand; and 5 = very high relevant capacity/demand.

In this study, we adopted a semi-quantitative approach, which is acknowledged as the ES matrix approach to quantify the ES balance in Changchun City for 1990, 2000, 2010, and 2020. Because of differences in the actual geographic location and LULC classification systems, the original ES matrices of supply and demand were further adjusted by collecting and reviewing previous studies on ES balance and interviewing a group of experts to re-score the matrix entries applied in this study [5,8,28,32]. Eventually, the ES types were divided into three categories consisting of regulating services (9 types), provisioning services (11 types), and cultural services (3 types), in which the supply capability and relative demand of the aforementioned LULC (Figure 2) were assessed on a scale ranging from 0 to 5 according to Burkhard’s studies and the actual regional situation, in which 0 represents no relevant supply capacity/demand and 5 represents a very high relevant supply capacity/demand [5,33]. The ES supply and demand matrices are shown in Figure 3, and the ES supply index (*ESSI*), demand index (*ESDI*), and balance index (*ESBI*) were calculated using the following equations:

(1) $$ESSI_{t}=\sum_{b=1}^{m}\sum_{a=1}^{n}\left(LUA_{a,t}\times S_{ab}\right)/\sum_{a=1}^{n}LUA_{a,t}$$

(2) $$ESDI_{t}=\sum_{b=1}^{m}\sum_{a=1}^{n}\left(LUA_{a,t}\times D_{ab}\right)/\sum_{a=1}^{n}LUA_{a,t}$$

(3) $$ESBI_{t}=\sum_{b=1}^{m}\sum_{a=1}^{n}\left(LUA_{a,t}\times B_{ab}\right)/\sum_{a=1}^{n}LUA_{a,t}$$

(4) $$B_{ab}=S_{ab}-D_{ab}$$

where $S_{ab}$, $D_{ab}$, and $B_{ab}$ represent the supply, demand, and balance matrices of the *b*th ES category of the *a*th LULC type, respectively. $LUA_{a,t}$ is the area of the *a*th LULC type at time *t*. Finally, *n* and *m* are the number of LULC types and ES categories, respectively, in this study.

#### 2.3.2. Assessment of Land Use Conflict

It is generally believed that land use conflict refers to the disharmonious and imbalance state disturbed by the intensification of anthropogenic processes and deterioration of natural conditions, the essence of which is the haphazard development process of land-centered resources at a variety of spatial and temporal scales, explicitly manifesting for social–resource conflict, eco-environmental conflict, and spatial conflict [22]. Since it is not only a miniature of the social-ecological system but also the geographical phenomenon with spatial heterogeneity, we conducted and measured land use conflict through the complexity–fragility–instability dimensions of the land system at the grid level based on previous studies [22,34]. The spatial comprehensive conflict index (*SCCI*) can be expressed as follows:

(5) SCCI=CI+FI+ISI

where *CI*, *FI*, and *ISI* represent the complexity index, fragility index, and instability index of land use conflict, respectively.

(1) Complexity index (*CI*)

The area-weighted mean patch fractal dimension (AWMPFD), which can reflect the complexity of land use structure and landscape patterns, was used to measure the *CI* of land use conflict, and its formula is as below:

(6) $$CI=\sum_{i=1}^{u}\sum_{j=1}^{v}\left[\frac{2\ln\left(0.25P_{ij}\right)}{\ln\left(a_{ij}\right)}\left(\frac{a_{ij}}{A}\right)\right]$$

where $P_{ij}$ and $a_{ij}$ are the perimeter and area of patch *j* in land use type *i*, respectively. *A* is the grid size of the fishnet, *v* is the total number of patches in the grid, and *u* is the number of land use types. In general, a higher *CI* value often denotes more complex and mixed landscape patterns and more intense land use conflict.

(2) Fragility index (*FI*)

The *FI* embodies the response capability of the land use system to internal disturbances and external pressures, as a higher fragility degree tends to have a higher level of land use conflict. Fragility is deemed to be closely related to the dynamic transition of land use types [35]. In this study, the transfer-out rates of construction land, cultivated land, forest, grassland, water, wetland, and unused land from 1990 *to* 2020 were 13.87%, 6.94%, 11.87%, 35.05%, 31.42%, 37.32%, and 24.62%, respectively. Therefore, we assigned the fragility scores of seven land use types, ranked in ascending order, as cultivated land (1), forest (2), construction land (3), unused land (4), water (5), grassland (6), and wetland (7). The *FI* was calculated as

(7) $$FI=\sum_{i=1}^{u}F_{i}\times \frac{a_{i}}{A}$$

where $F_{i}$ is the fragility score of land use type *i* adopted for this study, $a_{i}$ is the total area of land use type *i*, *A* is the grid size of the fishnet, and *u* is the total number of land use types.

(3) Instability index (*ISI*)

According to Equation (8), landscape fragmentation with patch density was used in this study to estimate the *ISI*, where the higher the index value, the more fragmented the landscape in the land system, indicating the lack of landscape stability and thus increasing land use conflict:

(8) $$ISI=\frac{N_{i}}{A}$$

where $N_{i}$ is the patch number of landscape type *i* and *A* is the grid size of the fishnet.

In addition, given that different dimension indices have positive impacts on land use conflict, the range transformation method was used to normalize the calculation results of the *CI*, *FI*, and *ISI* to calculate the *SCCI*.

### 2.4. Evaluation of the Coupling Coordination Degree of ES Balance and Land Use Conflict

The coupling coordination degree is a physical concept that refers to the interaction of different systems under their own or external effects [29]. It is a concept that can specify land system states and determine the development direction and intensity between the ES balance system and the land use conflict system at different times. Therefore, a coupling coordination degree measurement model was constructed to evaluate the coupling relationship and degree of coordination and consistency based on the normalized values of land system adaptation and conflict. The calculation formulae are as follows:

(9) $$D=\sqrt{C\times T}$$

(10) $$C={\left\{\left(U_{1}\times U_{2}\right)/{\left(\frac{U_{1}+U_{2}}{2}\right)}^{2}\right\}}^{1/2}$$

(11) $$T=\alpha U_{1}+\beta U_{2}$$

where *D*, *C*, and *T* represent the coupling coordination degree, coupling degree, and comprehensive coefficient of the coordination degree, respectively. $U_{1}$ and $U_{2}$ are the normalized index values of ES balance and land use conflict. α and β can be interpreted as the contribution of the two systems, and their relative magnitudes basically do not affect the spatial trends in the coupling coordination degree. In this study, we believe that the level of ES balance is as important as land use conflict, so both values were set to 0.5. According to existing research, the classification of coupling coordination level types is shown in Table 1 [36].

## 3. Results and Analysis

### 3.1. Spatio-Temporal Evolutions of the Supply, Demand, and Balance of ES from 1990 to 2020

**Figure 4:**
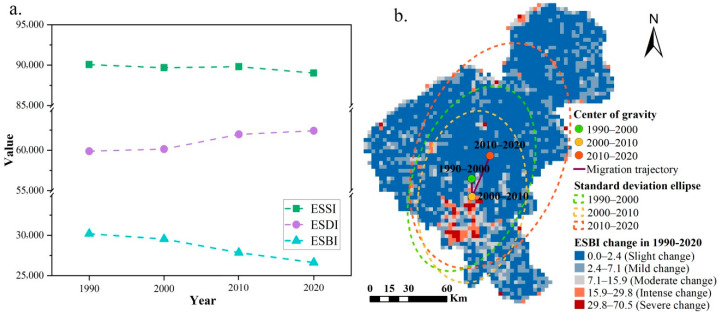
Overall trends of the ESSI, ESDI, and ESBI (**a**) and spatial migration map of the absolute ESBI changes (**b**) from 1990 to 2020.

Based on the ES supply–demand matrices and LULC data, we quantified and visualized the spatio-temporal characteristics of ES balance from 1990 to 2020. Our results showed that the ESBIs of Changchun City in 1990, 2000, 2010, and 2020 were 30,190.0, 29,532.2, 27,847.6, and 26,617.6, respectively. The estimated ES supplies evidently surpassed their demands, especially in 1990 (Figure 4a). Overall, there was no significant change in the ES supply, while the ES demand continued to rise, demonstrating an obvious downward trend in the ES balance, with a decline rate of about 11.8%.

**Figure 5:**
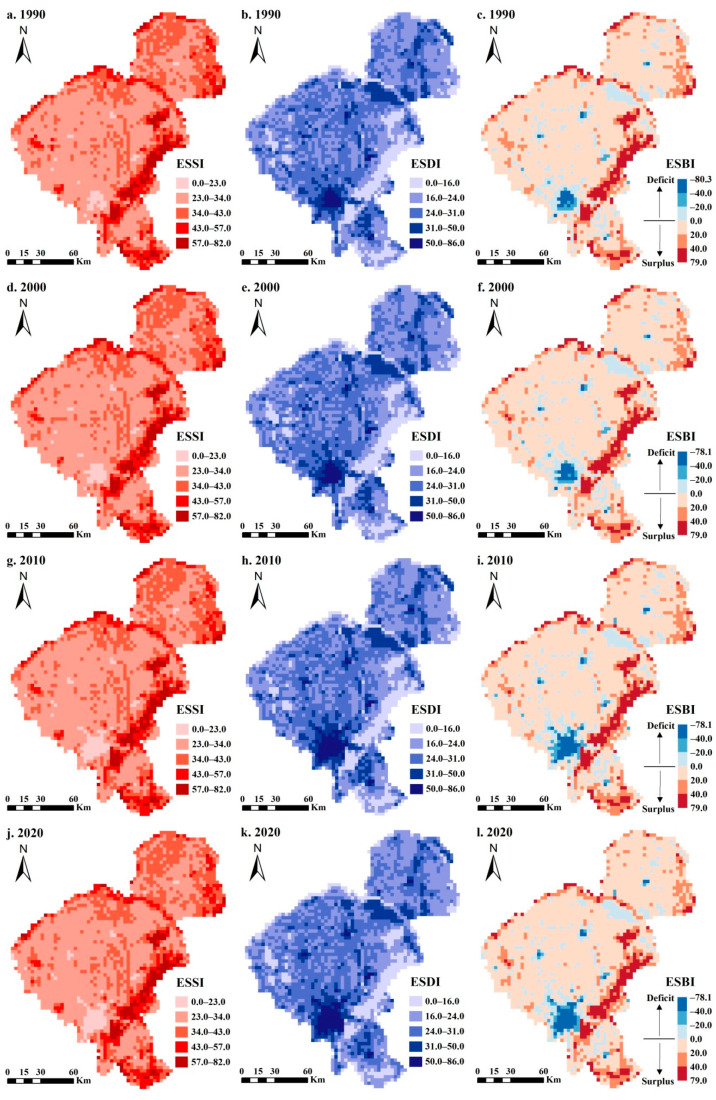
(**a**–**l**) Spatial distributions of the ESSI, ESDI, and ESBI in Changchun City from 1990 to 2020.

The spatial patterns of the ESSI, ESDI, and ESBI for the four periods were essentially dictated by the LULC conditions (Figure 5). For ES supply, the spatial distribution of the ESSI ranged from 0.0 to 82.0 and was relatively high in the southeast compared to the northwest (Figure 5a,d,g,j). The higher areas were mainly concentrated in the eastern part of the study area, where forest land was widely distributed, and there was a continuously decreasing trend of in the ESSI in the southern built-up area because of the higher proportion of construction land. To a certain extent, the changes in the ESDI and ESSI exhibited opposite spatial distribution characteristics, whereas a high ES demand mostly occurred in northern and southwestern Changchun City (Figure 5b,e,h,k). Notably, the most dramatic expansion of the ESDI was in good accordance with the area where the ESSI significantly reduced during 1990–2020, indicating that the regional ES supply and demand tend to have opposite trends of deterioration and improvement. The ESBI showed a similar spatial variation pattern as the ESSI but was different from the ESDI (Figure 5c,f,i,l). Most of the study areas had positive ESBI values and ES surpluses, particularly for the eastern areas, which were characterized by a high forest cover. However, the areas with ES deficits, that is, the areas where ES demands surpassed their supply, were far smaller than the areas with ES surpluses and spatially paralleled those with a high ES demand. The absolute changes in the ES balance are shown in Figure 4b. During the period of 1990 to 2020, the ESBI changed significantly and was generally at a slight change level, with only a small portion of grids around the southern urban area presenting intense-to-severe changes. According to the gravity-center migration model and the standard deviation ellipse model, the spatio-temporal migration direction of the ES balance was mainly from north to south and then to the northeast, where the spatial transition between 2010 and 2020 was more variable than that in the 2000–2010 period.

### 3.2. Spatio-Temporal Patterns of Land Use Conflicts from 1990 to 2020

**Figure 6:**
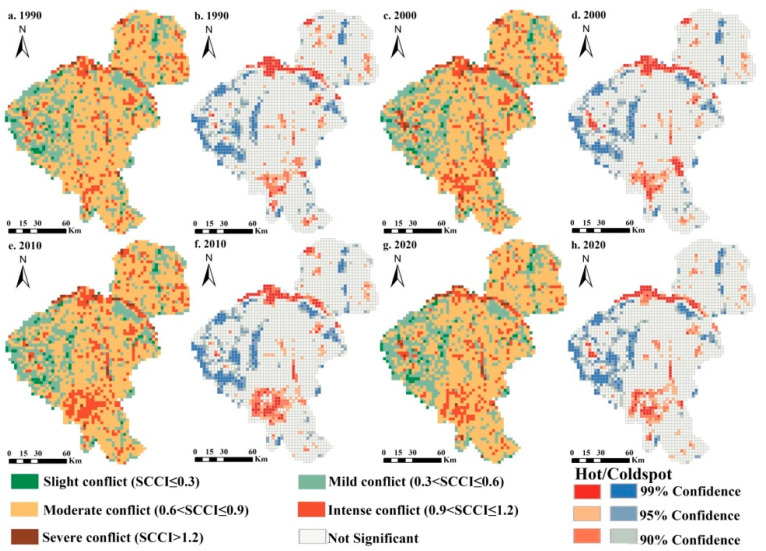
Spatio-temporal patterns of land use conflict in Changchun City from 1990 to 2020. (**a**,**c**,**e**,**g**) The evaluation results of land use conflict in 1990, 2000, 2010, and 2020, respectively; (**b**,**d**,**f**,**h**) the hotspot analysis results of land use conflict in 1990, 2000, 2010, and 2020, respectively.

As mentioned earlier, the land use structure of Changchun City underwent obvious dynamic transformations during the study period. We measured the spatial land use conflict by calculating the SCCI values and categorized the conflict types by comparing the measured values through the equal interval classification method, of which spatio-temporal characteristics are presented in Figure 6. The spatial pattern of regional land use conflict varied significantly, but the changes were minimal at each stage. In general, land use conflict was higher on the eastern side of the study area and was lower on the western side. With respect to different conflict types, moderate conflict was identified as being widely distributed all the time; other than that, mild conflict and intense conflict were also considered to be the principal spatial conflict types, of which the former primarily occurred in the cold spots of the SCCI and was concentrated largely in the western and northern areas, while the latter was composed of the hot spots of regional land use conflict, demonstrating significant ring clusters and belt agglomeration patterns in the south and central areas. Obvious regional differences were observed in southern urban development areas, where the spatial pattern of land use conflict has altered considerably. These hot spots expanded noticeably from 2000 to 2020.

**Figure 7:**
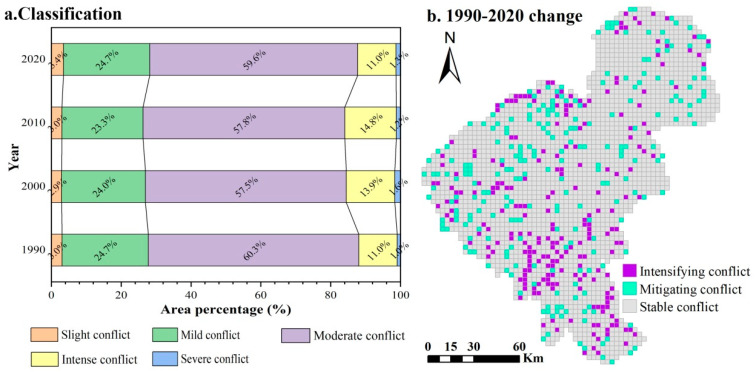
Types of land use conflict (**a**) and spatial patterns of conflict changes (**b**) from 1990 to 2020.

Over the study period, the average SCCI values were about 0.7, indicating that the overall land use conflict has been at a moderate conflict level. There was no significant change in the structure of land use conflict (Figure 7a), with moderate conflict accounting for nearly 60.0% of the study area and dominating, followed by mild conflict and intense conflict, and slight conflict and severe conflict being the least. The total proportion of intense and severe conflicts showed a tendency to increase and then briefly fall, which reached 12.3% in 2020, with an increase of 0.3% compared to that in 1990. The spatial variation of the SCCI revealed the stability and change direction of the land use conflict structure over the past three decades (Figure 7b). It is evident that the spatial patterns of land use conflict in the study area remained basically stable, and mitigating and intensifying conflicts were sporadically distributed, accounting for 81.2%, 9.5%, and 9.3% of the region, respectively. Land use conflict changes in urban sprawl areas showed a significant increasing trend during the study period, while a similar trend could be found in land use conflict hot spots.

### 3.3. Coupling Coordination Characteristics between ES Balance and Land Use Conflict

**Figure 8:**
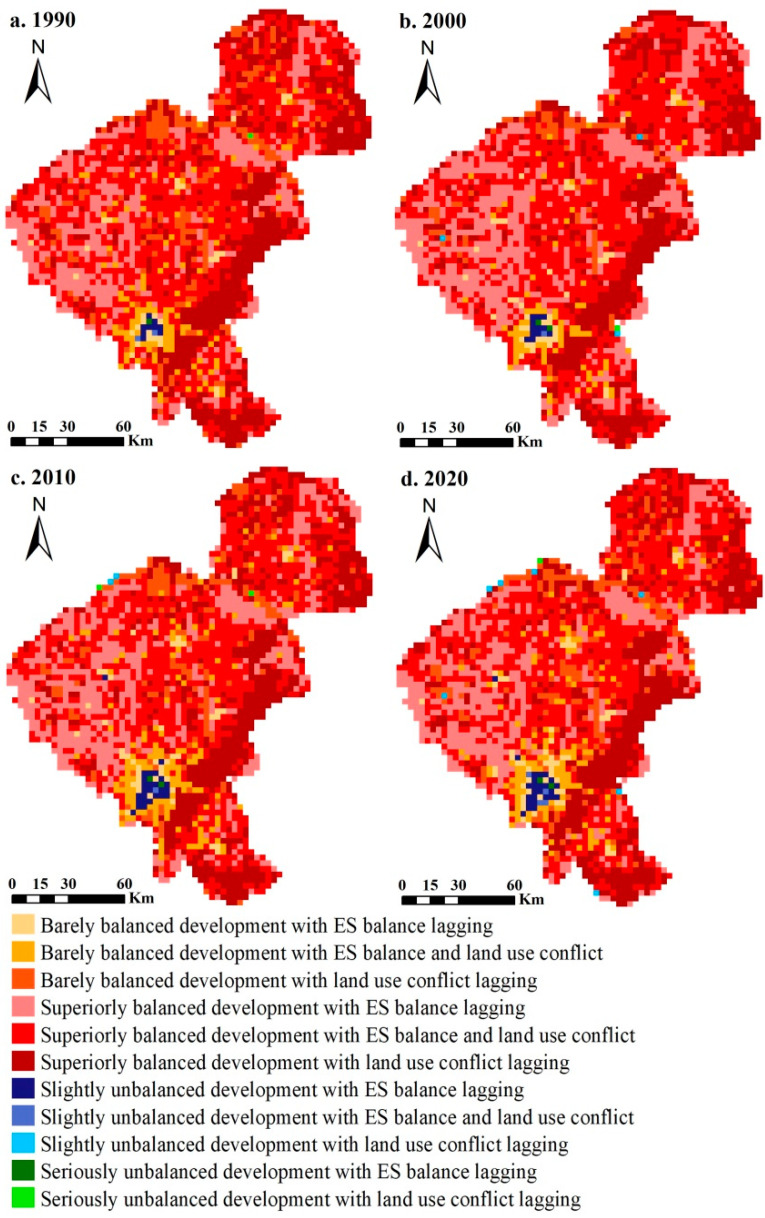
Spatial distribution of the coupling coordination degree of ES balance and land use conflict in 1990 (**a**), 2000 (**b**), 2010 (**c**), and 2020 (**d**), respectively.

The coupling coordination degree between ES balance and land use conflict is a nonlinear response to the complex interlacing and spatio-temporal variation characteristics of the internal adaptation support and external conflict pressure in a land system. As illustrated in Figure 8, the regional coupling coordination relationship has always been dominated by a balanced development pattern throughout the research period, with few unbalanced development areas, and the comprehensive level has been maintained at the superiorly balanced development stage. This indicates that there was an obvious interactive driving effect between ES balance and land use conflict. The coupling coordination intensity was characterized by significant spatio-temporal diversity. It is seen that the development pattern was characterized by a “high in the west and east, and low in the middle” balanced distribution, except that unbalanced development areas were located in the southern part of the city. Due to the external expansion of the built-up areas, the agglomeration characteristics of the slightly unbalanced development areas were gradually enhanced, along with the barely balanced development belt surrounding and experiencing a circle-layer growing trend. From the perspective of spatial variations of coupling coordination types, we could track the land system states, in which the urban center tended to be in a stage of uncoordinated development and the ES balance was blocked. Conspicuous regional variability can be observed in the barely balanced development regions and superiorly balanced development regions as well. According to the results, there were widespread balanced development areas with the synchronized type of ES balance and land use conflict in the middle of the study area, where the land system developed coordinately and synchronously. The spatial pattern of the lagging ES balance type is partly consistent with the cold spots of land use conflict. In addition, the areas with lagging land use conflict were represented by the eastern forest region, where the ES balance was at a high surplus level, meaning that the land system in this area seems to achieve high-level coordination and orderliness.

## 4. Discussion

### 4.1. How Do ES Balance and Land Use Conflict Track Land System States?

**Figure 9:**
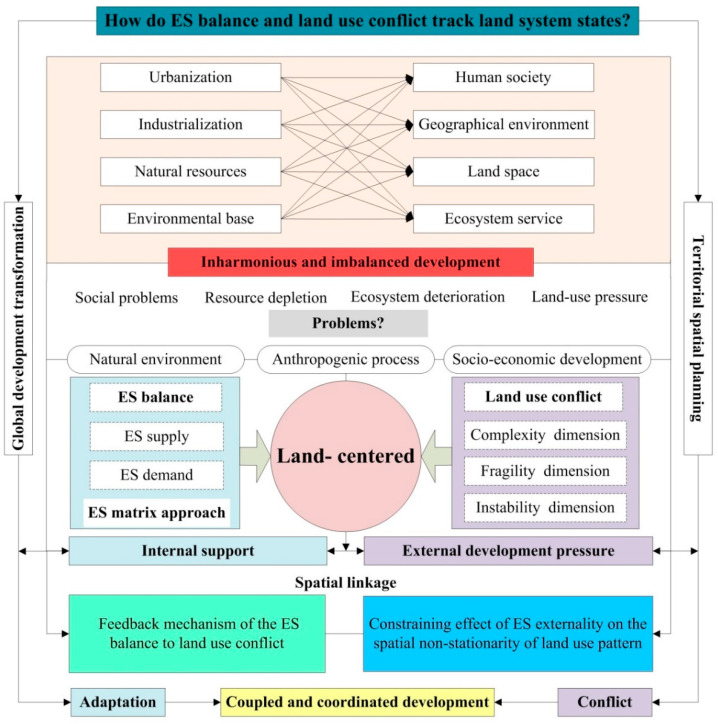
The logic diagram of combining ES balance and land use conflict to track land system states.

The spatial heterogeneity of ES supply and regional land development often directly affects the ecosystem structure and leads to the supply of ESs varying greatly among regions that do not necessarily coincide with the geographical location of high-socio-economic-demand areas, and a reduced ES balance can also generate inequitable access to resource benefits [9]. This study clarified the land system states in terms of the internal support and external development pressure of the land system by understanding the interactive relationship between ES balance and land use conflict (Figure 9). On the one hand, it refers to the feedback mechanism of the ES balance to land use conflict, mainly in terms of the threatening effect of urban land use on the ecosystem changes. In the study region, the imbalance between the supply and demand of ESs was significant, and the acceleration of urbanization exacerbated the spatial deficit of the ES balance in the central city over time. The transformation of the urban and rural regional landscape, namely spatial urbanization, is principally reflected in the increase in the urban construction density and the expansion in the territorial scale. The process directly leads to the growth of land use conflict, which has a profound influence on the scale and morphological resilience of land use, making the ecosystem suffer from disturbances and impacts that cannot be completely avoided. The mechanism of the ecological disorder process caused by land use conflict on the ES balance can be summarized as follows: As a medium of spatial expansion, land resource transfer, especially the irrational allocation of urban construction land and ecological space, will weaken the permeability of ecological elements in the built-up environment, while acting indirectly on the migration of the level of the ES balance as the core driving factor putting stress on the supply and demand of ESs. A given urban space or landscape is the spatial carrier of urban ecosystem function, and the optimization of the urban spatial structure should be fully promoted to guide interactions between ES supply and functional requirement, ES balance, and land development. Meanwhile, while emphasizing the leading role of land urbanization, it is still necessary to continuously promote the effective implementation of ecological compensation so as to realize the supply–demand flow of ESs [37,38].

On the other hand, such an interactive driving effect can be expressed in the constraining effect of ES externalities on the spatial non-stationarity of urban land use. The human demand for eco-environment services constitutes a prerequisite for land system sustainability. Population expulsion, location selection, social change, and capital exclusion, as the primary causes of human–land contradiction, are the external pressures of ecosystem degradation caused by land use conflict [29]. Since urbanization is materially rooted in ES functions, the decline in the internal support of ESs, in turn, restricts the development of the urbanized land system [10]. From 1990 to 2020, the ESBI of the southern built-up area showed a strong negative pattern, together with its outer layer evolving intensively. This phenomenon directly reflected that the internal support of the land system in urban and rural–urban transformation areas was relatively weak. It is worth noting that the land use conflict eventually manifested as the hot spots outpacing the urban development boundary as a result, which is consistent with the aforementioned areas. The unsound impacts of rapid urbanization, including the blind expansion of urban land, and the increased spatial complexity, fragmentation, and non-stability of landscape patterns resulting from low-density land use patterns have damaged land development and appear to be the cause of urban land use conflicts [34]. These findings seem to confirm the notion that the intensive exploitation of land used for urban development is often closely linked with the ecosystem structure and process.

In this context, how to balance ecosystem conservation with the actual demands of human livelihoods in the land system is a challenge facing considerable threats. Reasonable spatial regulation is often regarded as an effective tool to reduce the damage of land use conflict on the ES balance and access ES functions. The Chinese government has carried out a series of policies/projects, such as the Grain for Green project, the permanent basic farmland protection policy, and the landscape consolidation programs, to protect and restore the ecosystem and alleviate the increasing conflict intensity caused by rapid land-use changes [23]. However, it seems that only by coordinating the use of ES balance and land use conflict can a sustainable development goal in the land system be achieved and thus enable the avoidance of both the “degradation and conservation traps” [8,39]. Therefore, it is particularly important to combine regional development differences, optimize the layout of urban development and green spaces, and improve the quality and stability of the ecosystem, thereby achieving a win-win outcome that mitigates ecosystem degradation and guarantees land development.

### 4.2. The Reality and Potential of Coupling Coordination Development for Reconciling Urban Development Issues

Owing to the more diverse and dynamically transformed land use patterns around the city, both ES balance and land use conflict were higher in the urban periphery than in the center [40]. Indeed, as a systematic issue, urban development is intricately related to the interactions of ES balance and land use conflict, and understanding the coupling coordination relationships between them may help us to manage and reconcile these conflicts over the land use structure and ecosystem conversion. Based on our analysis, we found that the coupling coordination degrees of the adaptation–conflict system were relatively high in Changchun City, except for the southern urban area, which represented obvious constraint, feedback, coercion, and adaptability relationships. Moreover, the land system states of the main urban area and its surrounding areas can be divided into four stages: the low-level coordinated development stage (barely synchronous balanced development), the high-level coordinated development stage (superiorly synchronous balanced development), the antagonism stage (slightly synchronous unbalanced development), and the break-in stage (lagging ES balance or lagging land use conflict). Even though the spatio-temporal patterns of the coupling coordination degree showed a city-oriented and rating-circle structure ascending toward the urban periphery, there were different degrees of regressions with land use development. Due to the expansion and relocation of the trade-off development structure, the urban center, as a major growth pole of *Changchun Economic Circle Planning*, is stepping into a new break-in stage, in which the construction demand for “synergy and integration” of industrial development integration and eco-environmental protection integration has put forward new requirements for the urban area with a high level of land use conflict and lagging ES balance.

It has also provided potential for future exploitation, redevelopment, and revitalization of rust belt cities in the postindustrial era. With the expansion of the urban scale and the development of urban agglomerations, the urban demand for ESs is gradually transforming from local monomer facilities or specific projects to regional integration [41]. It has been proven that coupling coordination development theory can highlight the positive effects between multiple systems and can help monitor and depict the spatial distribution and dynamic evolution process of land system coupling coordination [30]. This paper used this theory to diagnose and track whether the “adaptation–conflict” system is in a coordinated development stage, expecting to provide a reliable reference for decision makers to guide land management, formulate spatially coordinated development policies, and solve the coupling and sustainable development issues between the man–land relationship and ecosystem health. Taking the opportunity of the reconstruction and optimization of urban spatial organization and regional green competitiveness, it is necessary to strengthen linkages between eastern ecologically sound areas and urban periphery areas where the land use conflict was evident and then foster regional integration, reallocate land use functions and construct a policy-led environmental justice system, ultimately achieving long-term urban and ecosystem sustainability within the context of regional development goals [34,42,43].

### 4.3. Limitations and Prospects

Concerning the limitations of data availability and research scale, there is still potential for further progress in coupling coordination development studies. In this study, land use conflict and ES balance were quantitatively evaluated from the perspective of the external pressure and internal support of the land system. The selection of indicators was typical but cannot guarantee comprehensiveness, which makes the assessed interactions of land use conflict and ES balance limited to within the available evaluation units; moreover, the spatial dependence and spatial heterogeneity among them were not discussed. The grid scale can characterize the slight regional differences intensively and thoroughly and find an ideal “point” to balance land development and ecological protection, thus better understanding the spatial correlation between subsystems in the problem of coupling and coordinated urban development. Nevertheless, in actual management and decision making, the values of external pressures should be specified and localized to ensure the practical value of the evaluation results. This study was primarily based on the following assumptions: (1) a land system that adapts to the external development demand is more sustainable (2) the higher the adaptability between systems, the more sustainable they are; and (3) the less internal conflict, the greater the sustainability. Therefore, subsequent studies will continue to focus on analyzing the structural characteristics of the land use conflict system and the influencing factors of the ES balance system and investigating the spatial heterogeneity and spatial spillover effects of the two systems through abundant index systems based on the identification of their spatial heterogeneity and spatial determinants to improve the accuracy of land system sustainability evaluation. Furthermore, there is a spatio-temporal lag in ES balance, and a multi-scenario simulation may be a feasible choice to realize the dynamic demand from the current status evaluation to tendency judgment.

## 5. Conclusions

In this study, ES balance and land use conflict were regarded as the internal support and external development pressure of the land system, respectively. We quantified and analyzed the spatio-temporal differentiation and evolution trends of ES balance and land use conflict in Changchun City, which is a typical rust belt region in China, and then explored their coupling coordination development relationships from 1990 to 2020. The findings are as follows: First, the overall ES balance demonstrated an obvious downward trend, and the spatial variation characteristics indicated that the higher ESBI values were mainly distributed in the eastern part of the study area. Second, the deficit ES balance areas (i.e., demand exceeding supply) were concentrated in the southern built-up area, and there was a significant expansion trend from 1990 to 2020. Third, the land use conflict was especially prominent in areas experiencing rapid rural–urban transformation where the hot spots also expanded noticeably. Finally, coupling coordination development analyses were used to track the development characteristics of the land system, and the results demonstrated that the study region has always been dominated by a balanced development pattern throughout the research period, except for the urban center, which tended to be in a stage of uncoordinated development, with the ES balance blocked. In addition, the agglomeration characteristics of the slightly unbalanced development areas were gradually enhanced, along with the barely balanced development belt surrounding and experiencing a circle-layer growing trend. The aforementioned results suggest that it is necessary to coordinate local and adjacent regions through regionally integrated efforts to alleviate the ES imbalance.

The findings of this study can provide a scientific reference for analyzing regional land system states and promoting revitalization and sustainable development in rust belt cities. Despite several limitations, investigating the coupling coordination development in a land system from an “adaptation–conflict” perspective is of great significance to direct future policy formulation about ecosystem conservation and spatial planning and eventually facilitate the achievement of a win-win strategy.

## Figures and Tables

**Table 1:** Classification of the coupling coordination degree [36] of ES balance and land use conflict.

Coupling Coordination Degree	First Grade	Relative Magnitudes of U_1_ and U_2_	Second Grade
0.7 < D ≤ 1.0	Superior balanced development	U_1_ − U_2_ > 0.1	Superiorly balanced development with ES balance lagging
		U_2_ − U_1_ > 0.1	Superiorly balanced development with land use conflict lagging
		0 ≤ |U_2_ − U_1_| ≤ 0.1	Superiorly balanced development with ES balance and land use conflict
0.5 < D ≤ 0.7	Barely balanced development	U_1_ − U_2_ > 0.1	Barely balanced development with ES balance lagging
		U_2_ − U_1_ > 0.1	Barely balanced development with land use conflict lagging
		0 ≤ |U_2_ − U_1_| ≤ 0.1	Barely balanced development with ES balance and land use conflict
0.3 < D ≤ 0.5	Slightly unbalanced development	U_1_ − U_2_ > 0.1	Slightly unbalanced development with ES balance lagging
		U_2_ − U_1_ > 0.1	Slightly unbalanced development with land use conflict lagging
		0 ≤ |U_2_ − U_1_| ≤ 0.1	Slightly unbalanced development with ES balance and land use conflict
0 < D ≤ 0.3	Seriously unbalanced development	U_1_ − U_2_ > 0.1	Seriously unbalanced development with ES balance lagging
		U_2_ − U_1_ > 0.1	Seriously unbalanced development with land use conflict lagging
		0 ≤ |U_2_ − U_1_| ≤ 0.1	Seriously unbalanced development with ES balance and land use conflict

## Data Availability

No applicable.
